# Literature Review of Postoperative Distal Radius Fracture Immobilization Recommendations

**DOI:** 10.7759/cureus.78349

**Published:** 2025-02-01

**Authors:** Alyssa Cevetello, Janae L Rasmussen, Hannah Sudhakar, Sydney Shindler, Rachel Yim, Rafay Hasan, Brandon Baek

**Affiliations:** 1 Osteopathic Medicine, Touro College of Osteopathic Medicine, Middletown, USA; 2 Orthopedic Surgery, Valley Consortium for Medical Education, Modesto, USA; 3 Osteopathic Medicine, University of New England College of Osteopathic Medicine, Biddeford, USA; 4 Osteopathic Medicine, Texas College of Osteopathic Medicine, Fort Worth, USA; 5 Osteopathic Medicine, A.T. Still University School of Osteopathic Medicine in Arizona, Mesa, USA

**Keywords:** distal radius fractures, geriatric fracture, hand and wrist surgery, postoperative immobilization, postoperative outcomes, wrist fracture, wrist splint, wrist surgery

## Abstract

Distal radius fractures (DRFs) are a commonly treated injury in orthopedics. DRFs have a high incidence across patient demographics, including pediatrics, young patients in high-energy trauma, and geriatric patients in low-energy trauma. While DRFs occur across a large range of age groups, they are especially consequential in geriatric patients with osteoporosis. Management of DRFs has extensive variability, ranging from conservative casting to surgical interventions, such as open reduction and internal fixation surgical procedures. The diversity of treatment options for DRFs is due to a consideration of factors, such as fracture characteristics, time to presentation with an orthopedic surgeon, age of the patient, and medical comorbidities of the patient. Despite being a common fracture type, there remain discrepancies in the non-pediatric literature regarding postoperative recommendations, such as the timing and methods of immobilization. There is also debate regarding whether postoperative immobilization in adult DRFs has clinical benefit. Some of this variability depends on the type of fixation utilized, such as a volar locking plate, dorsal locking plate, and dorsal wrist-spanning fixation. This literature review examines recommendations and outcomes of postoperative splinting (supination, pronation, or neutral rotation of the forearm) versus removable wrist brace versus soft dressings only for DRFs with both intra-articular and extra-articular fracture patterns with operative fixation. Postoperative care is imperative to study as it carries long-term effects on patients’ quality of life, as their range of motion and strength can be dictated by the methodology of this care. Studies have been conducted comparing the outcomes of early mobilization versus prolonged immobilization after surgical intervention. This literature review analyzes these studies to understand which methods carry better outcomes with respect to the range of motion and quality of life of patients for operatively treated DRFs in non-pediatric patients.

## Introduction and background

Introduction

Distal radius fractures (DRFs) are one of the common fractures encountered in orthopedic practice. An estimated 25% of fractures within the pediatric population and 18% of all fractures within the geriatric population have an effect on the distal radius [1,2]. These fractures can be broadly classified as having intra-articular involvement or being extra-articular. Intra-articular fractures involve the radiocarpal joint, disrupting the cartilage surface, and often require operative fixation to establish the accepted joint alignment tolerances. Extra-articular fractures do not directly involve the joint surface and are often treated conservatively with casting or splinting. The American Academy of Orthopaedic Surgeons (AAOS) has developed Appropriate Use Criteria (AUS) to help guide the treatment of DRFs by orthopedic surgeons [3]. While these recommendations provide helpful evidence-based guidance, there is still variability in the treatment options for patients. This can be attributed to variability in patient care considerations, such as fracture type, whether the patient had other injuries that could inhibit activities of daily living, hand dominance, patient age, patient activity level, access to follow-up care (e.g., no insurance), patient’s health literacy, and overall health status (e.g., comorbidities) [3]. Therefore, it is important for orthopedic surgeons to be aware of evidence-based recommendations when counseling patients with DRFs on their treatment options.

The prevalence of DRFs emphasizes their significance as a public health and scientific issue. These fractures often result from low-energy mechanisms, such as falls, and are strongly associated with osteoporosis in the geriatric population [4]. However, in younger individuals, the fractures often result from high-energy trauma, such as motor vehicle accidents and sports injuries [5]. DRFs are also common in pediatric patients, although their management is different due to special bone-healing characteristics of their physes (growth plates) [6].

Despite advances in surgical techniques consisting of volar and dorsal locking plates, there remains no clear consensus regarding whether immobilization is needed for postoperative DRFs, and what, if any, kind of immobilization may be of benefit. Some important questions still remain to be addressed. These include whether or not longer splinting in supination, pronation, or unbiased positioning produces higher consequences, and whether detachable wrist braces or gentle dressings are sufficient. This variability underscores the call for further study of postoperative immobilization techniques with their impact on optimal management of DRF.

This review evaluates the different effects of various postoperative immobilization strategies for operatively treated intra-articular and extra-articular DRFs, including the evidence for splinting in different forearm positions, detachable wrist braces, and soft dressings. This review also examines whether there is clinical utility in postoperative immobilization to help summarize evidence-based recommendations regarding the postoperative care of DRFs.

Methods

Six reviewers independently assessed studies published between 2000 and 2024 using data sources including Google Scholar and PubMed. They were searched between November 15, 2024, and December 15, 2024, using keywords in combination with “distal radius fracture” and/or “wrist fracture”, “wrist immobilization”, “volar plating”, “dorsal plating”, “fracture(s)”, “cast”, “splint”, “guideline” and/or “management”, and “trauma”. For the purpose of this review, only studies related to non-pediatric operatively treated intra-articular and extra-articular DRFs were included. Studies were excluded if they were published before 2000, addressed pediatric populations, or did not address the purpose of this review. No meta-analysis was performed.

## Review

Types of immobilization for postoperative distal radius fractures

Postoperative immobilization techniques for DRFs have been an area of intrigue between providers. The choice of postoperative immobilization, whether through splinting, a removable wrist brace, or soft dressing, depends on several factors. These factors include the type of DRF, surgical approach, and patient-specific considerations, such as activity level and compliance with rehabilitation protocols. A common approach used involves the use of a splint for the initial two weeks after the operation, at which point patients are placed in a removable wrist brace. However, few studies have assessed the efficacy of specific rotational positioning within a splint in comparison to using a removable brace or soft dressing immediately postoperatively.

Splints can be designed in various positions, such as supination, pronation, or neutral. Advantages of splinting include greater rigidity, stability, and overall patient comfort with a reduced risk of reduction loss [7]. Potential disadvantages include greater material cost and less hand or wrist mobility, which can result in joint stiffness [7]. Supination places the forearm in a “palm up” position, pronation places the forearm in the “palm down” position, and a neutral placement limits the rotation of the forearm in either direction. Each position works to optimize soft tissue tension and maintain fracture reduction during the early phases of healing. Since optimal protocols for postoperative immobilization have not been established, techniques are largely based on surgeon preference. Where an orthopedic surgeon trained, whether they obtained a hand or upper extremity fellowship, and their current practice location are all potential influencing factors.

Recent studies continue to show a lack of consensus regarding the optimal positioning for splint immobilization, which can be attributed to the varied methodology of prior research, variations in the training of orthopedic surgeons, and variations in patient characteristics (e.g., geriatric with osteoporosis versus a comminuted DRF in a young patient). Hill et al. assessed 32 patients at two and six weeks postoperatively to measure patient-rated wrist evaluation (PRWE), Disabilities of the Arm, Shoulder, and Hand (DASH) scores, visual analog scale (VAS) pain scores, range of motion (ROM), and grip strength [8]. The study found no significant difference in patient measurements between the maximal supination (sugar tong) and no restriction of supination (volar) splint groups [8].

Other immobilization options are removable wrist braces, which provide a balance between immobilization and functionality. Removable wrist braces allow patients to remove the device for hygiene, comfort, or rehabilitation exercises. The use of a brace for stabilization immediately following surgery has gained interest in recent years. A study assessing the patient quality of recovery one week postoperatively found no significant differences between removable brace and traditional casting groups [9]. The study utilized a Quality of Recovery (QoR-15) assessment with an 11-point scale system to determine patient pain, physical well-being, physical independence, psychological support, and emotional state at set points during the first postoperative week [9]. The brace group reported increased pain during the first 24 hours postoperatively (p = 0.022), but differences in other domains were negligible [9]. Further studies with more protracted recovery outcomes are necessary to assess clinical outcomes and patient-reported outcomes with removable wrist braces compared to other methods.

Soft dressings are less restrictive in comparison to splinting and allow for earlier wrist motion. Dressings are traditionally thought to provide less support during acute postoperative care when used alone, leading to potential patient discomfort and risk of reduction loss. However, a recent study assessing the differences in outcomes with the use of plaster splinting versus soft dressing following operatively treated DRFs found favorable outcomes with the use of soft dressing for postoperative care [7]. By the final postoperative visit, the soft dressing group showed a statistically significant increase in ROM in flexion, extension, and supination in comparison to the plaster splinting group [7]. Additional significant improvements were noted in the soft dressings group for PRWE, DASH scores, and VAS pain scores [7]. Another study achieved similar results when assessing postoperative outcomes between a compression bandage group and a forearm splint group [10]. No significant clinical or radiological differences were reported between the groups for PRWE, VAS, ROM, and union time [10]. This supports ongoing research on considerably larger splints having no distinct advantage over soft dressings for postoperative care. In addition to reduced cost and application time, the soft dressings showed no increased risk of reduction loss, increased pain, or compromised ROM in comparison to plaster splinting. Avoiding immobilization in postoperative DRFs is becoming more commonly accepted in light of studies demonstrating improved early ROM and improved patient outcomes.

Variations in length of immobilization and weight-bearing status

An additional consideration in immobilization therapy following surgical treatment of DRFs is the duration of immobilization and weight-bearing status of the joint following fracture; these protocols for length of immobilization following DRFs vary. Some studies suggest that earlier mobilization may yield better outcomes than prolonged immobilization in both conservative and surgical settings [11]. One systematic review of nine randomized controlled trials found that patient outcomes were significantly better in patients treated with early mobilization of the wrist compared to those in the late mobilization group at six weeks postoperatively [11]. Outcomes were measured by DASH scores at several intervals following surgery, as well as through PRWE, grip strength, VAS scores, and wrist ROM [11]. However, in the long-term follow-up period, differences between the two groups diminished, and outcomes were ultimately similar between early and late mobilization groups [11]. These studies demonstrate that there may be benefits to early mobilization for patients who wish to return to work or other activities earlier. This is an important consideration, especially for younger patients, those who had multiple traumatic injuries, and those who are independent at baseline. Additionally, patient factors, such as health literacy and patient reliability with following instructions, may influence a surgeon’s postoperative plan. For example, a patient who demonstrates an understanding of instructions may be trusted to only have soft dressings and start earlier ROM.

Similarly, a randomized controlled trial assessed 81 patients with DRFs treated with open reduction and internal fixation (ORIF) with a volar locking plate [12]. Patients began postoperative care with three to five days of gentle active ROM wrist exercises in both groups [12]. Following this, one group of patients began an accelerated program two weeks post operation of passive ROM and strengthening exercises (utilizing wrist isometrics, isotonic strengthening, and gripping with putty) [12]. This group experienced significantly improved mobility, strength, and DASH scores compared to patients who began passive ROM and strengthening exercises at six weeks [12]. However, the authors note that these group differences diminished by 12 weeks postoperatively, suggesting that early mobilization may mostly provide a benefit for earlier return to function [12].

The benefit of early mobilization has been supported by other studies, one being a randomized controlled trial assessing 133 patients with a DRF treated with ORIF utilizing a volar locking plate [13]. This study found that in patients who were prescribed one, three, or six weeks of postoperative immobilization, the one-week and three-week groups had better PRWE and ROM scores (flexion and extension) than the six-week group at the six-week mark postoperatively [13]. At 12 weeks and six months following surgery, the groups no longer exhibited significant differences in these outcomes, bolstering the suggestion that the benefit of early discontinuation of immobilization may be limited to the short-term effect of earlier regain of function [13].

A randomized controlled trial assessed 60 patients with an isolated DRF treated with a single fixed-angle volar plate and screws [14]. Patients were assigned to either an early motion (mobilization of the wrist joint within two weeks) or late motion (mobilization of the wrist joint at six weeks) group, and it was found that there were no significant differences between the groups [14]. This deduction was determined in terms of ROM, grip strength, radiographic evaluation, pain ratings, modified Gartland and Werley scores, Mayo wrist scores, and DASH scores [14]. The authors suggest that while wrist mobilization within two weeks after fixation of a DRF with a volar locking plate is likely safe, the data did not indicate that this early mobilization improves outcomes with respect to ROM, function, or health status [14]. However, participants in this study were not closely followed to assess adherence to the two groups’ treatment protocols, but rather “early motion” participants were taught active and active-assisted wrist motion exercises earlier than their late motion counterparts [14]. Concurrently, “late motion” participants were immobilized with removable splints, and not solid casts [14]. The patient's compliance with the prescribed treatment protocols was not measured or reported [14]. Additionally, evaluations of wrist function were conducted only at three months and six months post operation [14]. As indicated by Deng et al., Brehmer & Husband, and Watson et al., the three-month mark may be too late in the healing process to highlight the differences between early and late motion groups [11-13].

Similarly, a randomized controlled trial assessed 95 patients with DRFs treated with ORIF utilizing a volar locking plate [15]. This study found that groups with early mobilization (removable orthosis and daily non-weight-bearing wrist exercises) and late mobilization (standard dorsal plaster cast for two weeks, followed by removable orthosis and wrist exercises) did not report any significant differences in the ROM, grip strength, or in the DASH scores at four weeks, three months, six months, and 12 months postoperatively [15]. Sørensen et al. hypothesized that this finding could, in part, be due to the wrist exercises conducted by participants in the early mobilization group not being sufficiently weight-bearing [15]. This suggests that a more aggressive treatment protocol, such as that utilized in the randomized controlled trial by Brehmer & Husband, could yield better results in the early mobilization group [15].

Additionally, with more than 50% of patients with a DRF still employed, the physical restrictions imposed by reduced ROM, duration of sick leave, and impacts on quality of life can have significant socioeconomic effects on patients and their well-being [16]. Thus, the literature at present suggests that care should be taken to limit the duration of immobilization following operative treatment of DRF. Patients may experience improved and faster regain of function of their affected limb with an adequately aggressive exercise regimen initiated earlier in their postoperative period.

A summary of the studies included in this review is provided in Table 1.

**Table 1:** Summary of clinical studies. DRF = distal radius fracture; DASH = Disabilities of the Arm, Shoulder, and Hand; ORIF = open reduction and internal fixation; PROMIS = patient-reported outcome measures; PRWE = patient-rated wrist evaluation; ROM = range of motion; VAS = visual analog scale.

Author(s) & number of patients	Variables evaluated	Outcomes	Clinical importance
Poiset et al. [7] (N = 139)	ROM, PRWE scores, DASH scores, VAS pain scores, and radiographic data for loss of fracture reduction.	The study included patients receiving operative volar plate fixation for DRF. At three months postoperation, the soft dressing group showed improved ROM in extension (9.6˚), flexion (10.9˚), and supination (4.8˚) over the plaster splint group. Soft dressings showed significant improvements in PRWE, DASH, and VAS pain scores. No differences in radiographic outcomes were recorded.	Application of soft dressings following ORIF yielded improved functional outcomes at final follow-up in comparison to plaster splinting with no increased risk for loss of fracture reduction. The use of soft dressings is worth more investigation for DRFs.
Hill et al. [8] (N = 32)	ROM, grip strength, PRWE scores, DASH scores, and VAS pain scores.	The study included intra- and extra-articular DRF patients receiving operative volar plate fixation. At six weeks postoperatively, no significant differences occurred in patient outcome variables between the sugar-tong and volar splint groups.	Patient outcomes were similar regardless of splint immobilization position. Splint immobilization technique can be based on surgeon preference without compromising short-term function.
Sellbrant et al. [9] (N = 54)	Postoperative oral oxycodone use, quality of recovery, and perioperative time events in brace vs. cast groups.	The study involved patients receiving operative volar plate fixation for DRF. Both groups (brace & cast) demonstrated significant improvements in the quality of recovery, but higher pain was reported in the brace group. No significant differences between the two groups in oxycodone use.	Braces are adequate alternatives to casts for postoperative immobilization in DRFs as they offer similar quality of recovery.
Miró et al. [10] (N = 62)	VAS pain scores, PRWE scores, DASH scores, ROM, and complications.	The study included patients receiving operative volar plate fixation for DRF. No significant differences were found between patients immobilized with a soft bandage vs. with a splint three to twelve after DRFs.	A greater number of patients and follow-up visits are required to establish more universal protocols.
Deng et al. [11] (N = 596; 9 studies)	DASH scores, PRWE scores, pain rating, grip strength, ROM, and complications.	Systematic review with meta-analysis investigating early vs. late mobilization in patients with DRF treated surgically with open reduction and internal fixation. Early mobilization groups had better functional outcomes than the late mobilization groups at six weeks postoperatively. However, early mobilization groups did have a higher rate of implant loosening and/or fracture new displacement complication.	Early mobilization following ORIF of DRFs has a beneficial effect compared to late mobilization, especially at earlier stages of healing postoperatively.
Brehmer & Husband [12] (N = 81)	DASH scores, ROM, grip strength, and palmar pinch.	The study evaluates early vs. late mobilization in patients with DRF managed operatively with ORIF. Patients with an accelerated rehabilitation protocol, including earlier initiation of ROM and strengthening exercises, had better mobility, strength, and DASH scores from zero to eight weeks postoperatively compared to the standard rehabilitation group. However, these differences diminish by 12 weeks postoperatively.	Earlier initiation of range of motion and strengthening exercises following DRF treated with ORIF has a beneficial effect on functional outcomes at earlier stages of healing postoperatively. Long-term effects, however, were comparable between the two groups.
Watson et al. [13] (N = 133)	PRWE scores, pain rating, and ROM.	The study evaluates early vs. late mobilization in patients with DRF managed operatively with ORIF. When comparing patients treated with ORIF followed by immobilization for six weeks and patients with one to three weeks of mobilization postoperatively at the six-week mark, the mobilization group had significantly better PRWE scores and wrist flexion and extension active ROM. These differences diminished at 12 weeks and six months following surgery. Early mobilization also does not appear to carry an increased risk of adverse events compared to late mobilization.	Early mobilization following DRFs treated with ORIF yields an improved return to function with no increased risk of complication at earlier stages of healing postoperatively. However, long-term outcomes are similar between early and late mobilization groups.
Lozano-Calderón et al. [14] (N = 60)	ROM, grip strength, radiographic evaluation, Gartland and Werley scores, Mayo wrist scores, pain rating, and DASH scores.	The study evaluates early vs. late mobilization in patients with DRF managed operatively with single, fixed-angle volar plates and screws. There were no significant differences in functional outcomes at three or six months postoperatively between early and late mobilization groups following DRF treated with a single, fixed-angle volar plate and screws.	There is no difference in functional outcomes at three or six months postoperatively in patients encouraged to complete wrist exercises at two weeks following surgery compared to patients mobilizing at six weeks following surgery.
Sørensen et al. [15] (N = 95)	DASH scores, ROM, and grip strength.	The study evaluates early vs. late mobilization in patients with DRF managed operatively with ORIF. DASH scores improved significantly throughout the follow-up period of one year following DRFs treated with ORIF, and scores did not differ significantly between early and late mobilization groups. Similarly, ROM and grip strength were not significantly different between the groups. Groups also experienced comparable rates of postoperative complications.	Early mobilization following DRF treated with ORIF did not yield significantly better functional outcomes than late mobilization at several time points during a year postoperatively.
Zeckey et al. [17] (N = 50)	Modified Mayo wrist score, DASH, and ROM.	At six weeks, patients in the early mobilization group had better functional outcomes than patients in the splint group.	Early mobilization provides improved wrist function during early follow-up visits. However, postoperative care should depend on each patient's circumstances as splints can provide better physical protection.
Quadlbauer et al. [18] (N = 116)	Grip strength, radiographic findings, Quick DASH scores, PRWE scores, and VAS pain scores.	During the one-year follow-up, patients in the immediate mobilization group had higher ROM in extension and flexion, grip strength, and Mayo wrist score.	Immediate mobilization with formal physical therapy has a faster improvement in ROM and grip strength compared to patients placed in a cast.

Limitations

This review has limitations, including recognition of the heterogeneity of patient populations included in the cited studies, as well as the spectrum of different metrics used to assess patient outcomes. The lack of a narrowed definition of the patient population and a uniform measure of outcome may have contributed to the finding of a lack of consensus on postoperative immobilization of DRFs. Any attempt at constructing a clinical guideline may warrant more focused inclusion criteria for the type of DRF management as well as a tailored set of patient outcome measurements for objective comparison. This study is also limited in its ability to compare different populations, such as pediatric to geriatric, with DRFs fixed operatively as the accepted tolerances vary substantially. Additionally, there are limited studies assessing how factors like health literacy and social determinants of health may impact an orthopedic surgeon's decision on postoperative mobilization. This is important to investigate as decisions on DRF management involve considering factors outside of just radiographic findings. This study primarily focused on extra-articular DRFs, which does not consider variations in postoperative protocols to intra-articular DRFs. This literature review highlights the need for further research into this topic.

## Conclusions

This literature review indicates that there continues to be no consensus on the type of postoperative immobilization or length of immobilization recommended following operative fixation of intra-articular and extra-articular DRF. However, there are evidence-based recommendations supported by the American Academy of Orthopaedic Surgeons, along with literature to support earlier mobilization and avoidance of casts/splints in the postoperative period. While some studies have found early immobilization with either a removable brace or soft dressing with enrollment in a hand therapy program yields a faster return of ROM and strength, these findings tend to equalize with the longer immobilization patients over time. The use of a removable brace or soft dressing also contributes to increased functionality earlier following surgery. However, this can be offset by the increased risk of subsequent injury depending on the patient’s daily activities. It is important to consider factors like the dominant extremity of the patient, their work and independence status, their ability to obtain follow-up care (e.g., occupational therapy), and the patient's comorbidities. The consensus of postoperative immobilization continues to rely more on provider and patient preferences instead of any specific differences in outcomes in patient ROM and quality of life. More studies are needed to assess outcomes in the DRF immobilization method, length of immobilization, and recommendations for different patient populations and fracture characteristics.
